# Prevalence and determinants of unintended pregnancies amongst women attending antenatal clinics in Pakistan

**DOI:** 10.1186/s12884-017-1339-z

**Published:** 2017-05-30

**Authors:** Muhammad Atif Habib, Camille Raynes-Greenow, Sidrah Nausheen, Sajid Bashir Soofi, Muhammad Sajid, Zulfiqar A Bhutta, Kirsten I Black

**Affiliations:** 1 Discipline of Obstetrics, Gynaecology and Neonatology, Central Clinical School, University of Sydney, Sydney, NSW 2006 Australia; 2 Women and Child Health Division, Aga Khan University, Karachi, Pakistan; 3 Sydney School of Public Health, University of Sydney, Sydney, NSW 2006 Australia

**Keywords:** Unintended pregnancies, Family planning, Contraceptive methods, London measure of unplanned pregnancies, Pakistan

## Abstract

**Background:**

Unintended pregnancies are a global public health concern and contribute significantly to adverse maternal and neonatal health, social and economic outcomes and increase the risks of maternal deaths and neonatal mortality. In countries like Pakistan where data for the unintended pregnancies is scarce, studies are required to estimate its accurate prevalence and predictors using more specific tools such as the London Measure of Unplanned Pregnancies (LMUP).

**Methods:**

We conducted a hospital based cross sectional survey in two tertiary care hospitals in Pakistan. We used a pre tested structured questionnaire to collect the data on socio-demographic characteristics, reproductive history, awareness and past experience with contraceptives and unintended pregnancies using six item the LMUP. We used Univariate and multivariate analysis to explore the association between unintended pregnancies and predictor variables and presented the association as adjusted odds ratios. We also evaluated the psychometric properties of the Urdu version of the LMUP.

**Results:**

Amongst 3010 pregnant women, 1150 (38.2%) pregnancies were reported as unintended. In the multivariate analysis age < 20 years (AOR 3.5 1.1-6.5), being illiterate (AOR 1.9 1.1-3.4), living in a rural setting (1.7 1.2-2.3), having a pregnancy interval of = < 12 months (AOR 1.7 1.4-2.2), having a parity of >2 (AOR 1.4 1.2-1.8), having no knowledge about contraceptive methods (AOR 3.0 1.7-5.4) and never use of contraceptive methods (AOR 2.3 1.4-5.1) remained significantly associated with unintended pregnancy. The Urdu version of the LMUP scale was found to be acceptable, valid and reliable with the Cronbach's alpha of 0.85.

**Conclusions:**

This study explores a high prevalence of unintended pregnancies and important factors especially those related to family planning. Integrated national family program that provides contraceptive services especially the modern methods to women during pre-conception and post-partum would be beneficial in averting unintended pregnancies and their related adverse outcomes in Pakistan

## Background

Unintended pregnancies (pregnancies that are mistimed or unwanted) are a significant public health concern globally [1]. Of the estimated 210 million pregnancies that occur throughout the world each year, about 38% are unintended [2]. Twenty-two percent of global unintended pregnancies end in abortion, many of which take place with unsafe techniques and/or in unsafe circumstances and about 18% end in unplanned births, placing a substantial burden on health systems [1, 2]. Most of the unintended pregnancies occur in developing countries largely due to poor literacy and lack of knowledge and access to contraceptive methods [3, 4]. In these settings unintended pregnancies contribute significantly to adverse health, social and economic outcomes [4–8] and increase the risks of maternal death and neonatal, infant and child mortality [9].

Pakistan is a developing country where contraceptive prevalence remains low (35.4%) and the unmet need for family planning remains high (20.1%) contributing to high fertility rate (3.8 births/woman) and large numbers of unintended pregnancies [10, 11]. Annually about 2.25 million abortions are conducted in Pakistan and the national abortion rate is 50 per 1000 women (15-49 years) [12]. As abortion remains illegal, many of the procedures are undertaken in unsafe circumstances, leading to complications and adverse outcomes. Indeed in 2012 over 62,000 women were treated for complications [13]. Unsafe abortion also contributes to maternal mortality in Pakistan [14–16].

The reported prevalence of unintended pregnancies in Pakistan is between 16-46% [10–13]. Pakistan demographic and health surveys (PDHS) of 2006 and 2013 reported the prevalence of unintended pregnancies as 16% and 24% respectively [10, 11]. This estimate was based only on a single question with a dichotomous response on mistimed or unwanted pregnancy at the time of conception. Another study which estimated the prevalence of unintended pregnancies in Pakistan as 46% was based on an indirect modeling for unintended pregnancies from induced abortion rates [13]. These measures are not sufficient to accurately measure the burden of unintended pregnancies. However there is a more accurate, reliable and validated tool. The London Measure of Unplanned Pregnancies (LMUP) is a six item scale that has been widely used in both developed and developing countries [17–30].

Given the adverse impact of unintended pregnancies on maternal and neonatal morbidity and mortality, and the lack of available data, our aim was to investigate the prevalence of unintended pregnancy using the LMUP and examine the socio-demographic predictors in Pakistan.

## Methods

We conducted a hospital based cross sectional survey between January 2015 and April 2015 to achieve a sample size of 3000 women. We hypothesized that 40% of all pregnancies in the antenatal population in our study setting would be unintended, where the population is poor and there are high levels of illiteracy, little knowledge of contraception and where first pregnancies occur at a young age [11]. The sample size was estimated using a prevalence rate of unintended pregnancies of 40%, a confidence level of 95%, a design effect of 1.5 and a non-response rate of 10%. The data collection was carried out in two tertiary care hospitals, one is located in Karachi city and the other one is located at district Dadu of Pakistan. Both hospitals have an average attendance of 100 females per day. Two female research assistants were trained and employed at both sites for data collection. All pregnant women attending the antenatal care clinic were eligible for recruitment.

We developed a participant information sheet, consent form and an interview administered structured questionnaire. All material was translated into Urdu and then back translated into English to ensure the accuracy. The questionnaire was pre tested in an antenatal clinic that was not a study site. Women were given the participant information sheet to read, or when they were unable to read, the study was explained to them in Urdu by the research assistant.

The questionnaire comprised of three sections; section one used the standard questions from demographic and health survey questionnaire to ascertain the characteristics and socio-demographic information of the respondent. Section two contained information about past reproductive history and family planning and section three used the Urdu version of pregnancy intention scale (LMUP) to ascertain unintended pregnancies. In this section questions were designed such that each response to the six questions was scored out of two, and summed to give a final pregnancy intendedness score between zero and 12. The intention scores were divided into three groups: zero-three (unplanned), four-nine (ambivalent) and ten-twelve (planned).

Unintended pregnancy was the main outcome variable. Women with pregnancy intendedness scores less than 10 (including both ambivalent and unplanned pregnancies) were considered as unintended. The explanatory variables for analysis were informed by the literature and their availability in the dataset and are described in Tables 1 and 2. Variables were grouped into two categories; socio-demographic factors and women related factors. In the socio-demographic factors; age, residence, education and wealth index were considered. Age at marriage, gestational age, parity, birth interval, history of previous miscarriage or abortion, family planning knowledge, source of knowledge about family planning and family planning use were considered in the women related factors. Age at marriage was used as a proxy for age at first intercourse which is difficult to ask as it is a culturally sensitive question.

**Table 1:** Description of independent variables

Variables	Description
Socio demographic factors	
Area of residence	Urban and rural
Socio economic status of the household	SES was measured as quintiles of a linear index derived from household assets and utilities score, the wealth quintiles were divided into five (poorest, poorer, middle, richer, richest)
Women related factors	
Age	Categorized as <20 years, 20-24 years, 25-29 years and >30 years
Education	Years of education completed (illiterate/years of education)
Age of marriage	Categorized as = < 20 years and > 20 years, used as a proxy for age at first intercourse
Gestational age	Recorded in weeks and categorized as <28 weeks and 28 or more weeks
Parity	Defined as the number of previous deliveries and categorized as = < 2 times and > 2 times
Birth interval	Interval from one child's birth date until the next child's birth date and categorized as <12 months, 12-24 months and > 24 months
History of abortion or miscarriage	Any history of previous miscarriage and abortion and categorized as 1 = Yes and No = 2
Knowledge about family planning methods	Ever heard of any family planning method and categorized as 1 = Yes and No = 2
Use of family planning methods	Ever used any family planning method and categorized as 1 = Yes and No = 2

**Table 2:** London Measure of Unplanned Pregnancies (LMUP) scale

Question	Answer	Score
At the time of conception	Always use contraception	0
	Inconsistently us contraception	1
	Not use contraception	2
In terms of becoming a mother	Wrong time	0
	An OK time but not quite right	1
	Right time	2
Just before falling pregnant	Not intend to become pregnant	0
	Did not mind either way	1
	Intend to get pregnant	2
Just before falling pregnant	Not want a baby	0
	Have mixed feeling about having a baby	1
	Want a baby	2
Before falling pregnant had you and your partner	Never discussed children	0
	Discuss children but no firm agreement	1
	Agreed to pregnancy	2
Health actions before falling pregnant^a^	No action	0
	1 action	1
	2 or more actions	2

^a^Health Actions include iron folic acid supplementation, cessation or reduction in smoking, tobacco/ Pan/ Gutka/

beetle nut chewing and seeking medical advice

The ethical review committee of Aga Khan University granted ethical approval (Ref: 3710-Ped-ERC-15). Written informed consent was obtained from all participants. In cases where the woman was illiterate, consent was documented by a thumbprint on the consent form and confirmed by a signature from a literate witness. All the names and personal information regarding the participants were kept confidential and all identifying information was removed from analysis.

The data were analyzed using IBM SPSS version 19 [31]. Initially the scoring of the responses from the data extracted from LMUP was done and the prevalence of unintended pregnancy was calculated using data from all six of the questions in the pregnancy intention instrument. Family planning profile of the participants was also established and comparison of contraception knowledge and use between the data of this study and PDHS 2012 was carried out. Univariate analyses were run between socio demographic factors and women related factors. Degree of association was assessed using chi squared tests. The demographic characteristics with a *p*-value <0.25 were then examined using logistic regression analyses. In multivariate analysis adjusted odds ratios and 95% confidence intervals were calculated to determine the degree of association between associated factors and pregnancy intention.

In order to validate the Urdu version of the scale we conducted psychometric analysis of the Urdu LMUP. We assessed the proportion of missing data and considered item endorsement, with item response option endorsements of <80% considered to be acceptable [21–23, 26, 30]. To measure the reliability of scale, we evaluated the internal consistency by calculating the Cronbach’s alpha statistic using the standard cut off point of 0.7 and also looked at the corrected item-total correlations [21–23, 26, 30]. We also did Principal component analysis to evaluate the internal structure of the LMUP. The scale would be considered valid if all items load onto one component with an Eigenvalue larger than one (i.e. are measuring the same construct) [22, 26, 30].

## Results

A total of 3010 women were included in the analysis with a mean gestational age of 26 weeks at the time of recruitment. Overall, 1150 (38.2%) pregnancies in the antenatal population were unintended, of which 420 (13.9%) were ambivalent and 730 (24.3%) were unplanned. The remaining 1860 (61.8%) pregnancies were considered intended. The socio demographic profile and women related factors are documented in Table 3. The majority of women (69.5%) were aged more than 25 years, 51.6% were illiterate and half of the women lived in a rural area. Among the study population the two lowest and two highest wealth quintiles accounted for 40.2% and 40.1% respectively. We found that 55.0% of women were aged 20 years or younger at the time of their marriage, 46.1% had a parity of two or more and 53.6% reported a short birth interval of ≤ 12 months. Among study participants 17.4% reported a previous miscarriage or abortion.

**Table 3:** Frequency distribution of sociodemographic and women related variables

Variable	Description	N (%)
Pregnancy Intention	Unintended (Score <10)	1150 (38.20)
	Intended (Score >10)	1860 (61.7)
Area of Residence	Rural	1509 (50.1)
	Urban	1501 (49.9)
Wealth Index	Poorest	599 (19.9)
	Second	612 (20.3)
	Middle	593 (19.7)
	Fourth	604 (20.1)
	Richest	602 (20)
Pregnant women age	<20 years	135 (4.5)
	20-24 Years	783 (26)
	25-29 Years	1297 (43.1)
	> = 30 Years	795 (26.4)
Pregnant women’s education	Illiterate	1552 (51.6)
	Primary or less (1-5 years of schooling)	379 (12.6)
	Middle(6-8)	159 (5.3)
	Matric(9-10)	505 (16.8)
	Intermidiate & above (>10)	251 (8.3)
	Graduation and above (>12)	164 (5.4)
Pregnant women’s Age at marriage	≤20 Years	1656 (55)
	> 20 Years	1354 (45)
Gestational age	<28 weeks	1467 (48.7)
	> = 28 weeks	1541 (51.2)
Parity	<=2	1161 (53.9)
	>2	994 (46.1)
Birth Interval	≤ 12 months	1200 (53.6)
	>12 months	1038 (46.4)
History of Abortion/Miscarriage	Yes	523 (17.4)
	No	2487 (82.6)
Knowledge about Family Planning	No	306 (10.2)
	Yes	2704 (89.8)
Ever Used family planning methods	No	2004 (66.6)
	Yes	1006 (33.4)

**Fig 1:**
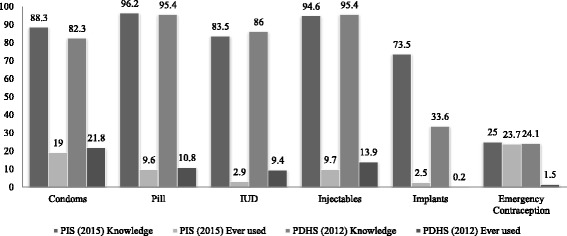
Comparison of contraception knowledge and use between survey for prevalence and determinants of unintended pregnancies among women attending antenatal clinics in Pakistan and PDHS 2012. **PIS~Pregnancy Intension Survey (Present study), **PDHS~Pakistan Demographic and Health Survey

The family planning profile is outlined in Table 4. Overall 89.9% women had knowledge about at least one of the contraceptive methods but only 33.4% reported using them. For modern methods 96.2% of women had knowledge of the pill, followed by injectables (94.6%), condoms (88.3%), intrauterine devices (83.5%), implants (73.5%), female sterilisation (60.9%), and male sterilisation (15.1%). However use of contraception remained low with the most commonly used being condoms (19%) followed by injectables (9.7%), the pill (9.6%), intra uterine device (2.9%), and implants (2.5%). For traditional methods only 14.5% and 34.5% of women had knowledge about the rhythm and withdrawal methods while 13.8% and 46.1% women reported using the rhythm method and withdrawal method respectively. Knowledge about emergency contraception was also low as only 25% of women were aware of it and only 23.7% reported having ever used it. The data regarding source of information for family planning revealed that health care providers (59.9%) are the main source of information followed by peers (22.2%), husbands (15.0%) and the media (1.4%). Our family planning data was consistent with the recent PDHS data and displays a notable contraceptive knowledge and practice gap (Fig. 1).

**Table 4:** Family planning knowledge, use and source of information

	Knowledge n (%)	Ever used n (%)	Health care providers n (%)	Media n (%)	Husband n (%)	Peers n (%)	Others n (%)
Any family planning method	2706 (89.9)	904 (33.4)	1621 (59.9)	39 (1.44)	406 (15.0)	602 (22.2)	36 (1.3)
Condoms	2658 (88.3)	505 (19.0)	1555 (58.5)	40 (1.5)	827 (31.1)	226 (8.5)	11 (0.4)
Pill	2896 (96.2)	278 (9.6)	1955 (67.5)	130 (4.5)	46 (1.6)	733 (25.3)	32 (1.1)
IUD	2513 (83.5)	73 (2.9)	1819 (72.4)	30 (1.2)	25 (1.0)	608 (24.2)	30 (1.2)
Injectable	2847 (94.6)	276 (9.7)	1987 (69.8)	80 (2.8)	31 (1.1)	715 (25.1)	34 (1.2)
Implants	2212 (73.5)	55 (2.5)	1712 (77.4)	13 (0.6)	20 (0.9)	453 (20.5)	13 (0.6)
Female sterilization	1833 (60.9)	04 (0.2)	1510 (82.4)	16 (0.9)	31 (1.7)	236 (12.9)	38 (2.1)
Male Sterilization	455 (15.1)	02 (0.4)	271 (59.6)	05 (1.1)	40 (8.7)	129 (28.4)	10 (2.2)
Emergency Contraception	753 (25.0)	178 (23.7)	386 (51.2)	05 (0.6)	93 (12.3)	267 (35.5)	03 (0.4)
Rhythm	436 (14.5)	60 (13.8)	199 (45.7)	04 (0.9)	125 (28.6)	102 (23.3)	07 (1.5)
Withdrawal	1038 (34.5)	478 (46.1)	158 (15.2)	03 (0.3)	654 (63.0)	195 (18.8)	28 (2.7)

Table 5 shows the univariate association between unintended pregnancy and the independent variables. Unintended pregnancy in Pakistani women was significantly associated with age < 20 years (OR 1.3 1.1-1.7), being poor (OR 1.8 1.3-2.3), being illiterate (OR 1.4 1.1-1.7), living in a rural setting (OR 1.5 1.1-1.8), having a pregnancy interval of ≤ 12 months (OR 1.8 1.3-2.9), having a previous history of miscarriage/abortion (OR 1.8 1.2-2.1), having parity of > 2 (OR 1.5 1.2-1.8), having no knowledge of any contraceptive method (OR 1.7 1.5-1.8) and never use of contraceptive methods (OR 1.2 1.1-3.8).

**Table 5:** Unadjusted association between unintended pregnancy and predictor variables, Pakistan 2015

Variable	Unintended pregnancy n (%)	OR	*p* value
Area of Residence			
Rural	670 (58.3)	1.5 (1.1-1.8)	<0.001
Urban	480 (41.7)	Ref	
Wealth Index			
Poorest	266 (23.1)	1.8 (1.3-2.3)	<0.001
Second	259 (22.5)	1.7 (1.2-2.1)	<0.001
Middle	244 (21.2)	1.6 (1.1-2.0)	<0.001
Fourth	199 (17.3)	1.1 (0.9-1.4)	0.311
Richest	182 (15.8)	Ref	
Pregnant women age			
<20 years	41 (3.6)	1.3 (1.1-1.7)	<0.001
20-24 Years	205 (17.8)	0.4 (0.3-0.5)	<0.001
25-29 Years	519 (45.1)	0.7 (0.6-0.8)	<0.001
> = 30 Years	385 (33.5)	Ref	
Pregnant women’s education			
Illiterate	552 (48)	1.4 (1.1 -1.7)	0.011
Primary or less (1-5 years of schooling)	153 (13.3)	1.1 (0.7-1.5)	0.769
Middle (6-8)	62 (5.4)	1.0 (0.6-1.6)	0.995
Matric (9-10)	216 (18.8)	1.2 (0.8-1.7)	0.398
Intermidiate & above (>10)	103 (9)	1.1 (0.7-1.6)	0.683
Graduation and above (>12)	64 (5.6)	Ref	
Pregnant women’s Age at marriage			
≤ 20 Years	648 (56.3)	1.1 (0.9-1.3)	0.248
> 20 Years	502 (43.7)	Ref	
Gestational age			
<28 weeks	534 (46.4)	1.2 (0.6-1.4)	0.071
> = 28 weeks	616 (53.6)	Ref	
Parity			
>2	528 (51.4)	1.5 (1.2-1.8)	<0.001
<=2	500 (48.6)	Ref	
Birth Interval			
≤ 12 months	521 (50.2)	1.8 (1.3-2.9)	0.003
>12 months	516 (49.8)	Ref	
History of Abortion/Miscarriage			
Yes	259 (22.5)	1.8 (1.2-2.1)	<0.001
No	891 (77.5)	ref	
Knowledge about Family Planning			
No	95 (8.3)	1.7 (1.5-1.9)	0.007
Yes	1055 (91.7)	ref	
Ever Used family planning methods			
No	657 (57.1)	1.2 (1.1-3.8)	<0.001
Yes	493 (42.9)	ref	

In the multivariate analysis (Table 6) being poor and having history of miscarriage/abortion no longer remained associated with unintended pregnancies but age < 20 years (AOR 3.5 1.1-6.5), being illiterate (AOR 1.9 1.1-3.4), living in a rural setting (AOR 1.7 1.2-2.3), having a pregnancy interval of = < 12 months (AOR 1.7 1.4-2.2), having a parity of >2 (AOR 1.4 1.2-1.8), having no knowledge about contraceptive methods (AOR 3.0 1.7-5.4) and never use of contraceptive methods (AOR 2.3 1.4-5.1) remained significantly associated with unintended pregnancy.

**Table 6:** Adjusted association between unintended pregnancies and of predictor variables, Pakistan 2015

Variable	Unintended pregnancy n (%)	AOR	*p* value
Area of Residence			
Rural	670 (58.3)	1.7 (1.2-2.3)	<0.001
Urban	480 (41.7)	ref	
Wealth Index			
Poorest	266 (23.1)	1.4 (0.9-2.2)	0.063
Second	259 (22.5)	1.7 (0.9-1.8)	0.068
Middle	244 (21.2)	1.6 (0.8-1.8)	0.087
Fourth	199 (17.3)	1.1 (0.8-1.4)	0.311
Richest	182 (15.8)	ref	
Pregnant women age			
<20 years	41 (3.6)	3.5 (1.1-6.5)	0.022
20-24 Years	205 (17.8)	1.0 (0.7-1.4)	0.937
25-29 Years	519 (45.1)	1.1 (0.9-1.4)	0.345
> = 30 Years	385 (33.5)	ref	
Pregnant women’s education			
Illiterate	552 (48)	1.9 (1.1-3.4)	0.025
Primary or less (1-5 years of schooling)	153 (13.3)	1.8 (1.0-3.1)	0.053
Middle (6-8)	62 (5.4)	1.8 (0.9-3.5)	0.100
Matric (9-10)	216 (18.8)	1.9 (1.1-3.2)	0.022
Intermidiate & above (>10)	103 (9)	1.5 (0.8-2.6)	0.195
Graduation and above (>12)	64 (5.6)	ref	
Parity			
>2	528 (51.4)	1.4 (1.2-1.8)	<0.001
<=2	500 (48.6)	ref	
Birth Interval			
≤ 12 months	521 (50.2)	1.7 (1.4-2.2)	<0.001
>12 months	516 (49.8)	ref	
History of Abortion/Miscarriage			
Yes	259 (22.5)	1.1 (0.7-1.8)	0.080
No	891 (77.5)	ref	
Knowledge about Family Planning			
No	95 (8.3)	3.0 (1.7-5.4)	<0.001
Yes	1055 (91.7)	ref	
Ever Used family planning methods			
No	657 (57.1)	2.3 (1.4-5.1)	<0.001
Yes	493 (42.9)	ref	

The psychometric analysis of the Urdu LMUP demonstrated relatively high internal consistency, with the Cronbach’s alpha score at 0.85 and the all item-rest correlations were 0.207 for item 1, 0.483 for item 2, 0.487 for item 3, 0.494 for item 4, 0.467 for item 5 and 0.235 for item 6.

**Fig 2:**
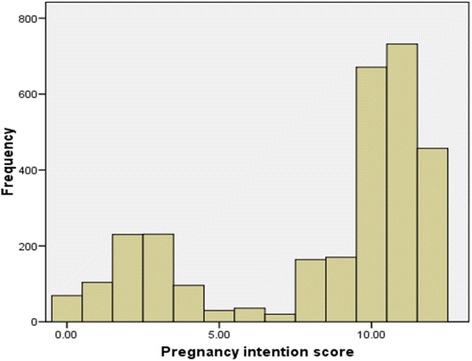
Distribution of Pregnancy intention score

We did not observe any missing data (Table 7). The LMUP score distribution was non-normal and the median score was 10 (inter-quartile range 5–11). The principal component analysis confirmed that all six items loaded onto one component (Eigenvalue = 3.81) and the six items component loadings were 0.146 for item 1, 0.865 for item 2, 0.870 for item 3, 0.902 for item 4, 0.815 for item 5 and 0.331 for item 6. We also report the full range of the LMUP scores (Fig. 2).

**Table 7:** Endorsement and response options for the LMUP scale

Endorsement of the PI items and response option		LMUP Pakistan	
Items	Category	n	%
At the time of conception	0. Always use contraception	642	21.3
	1. Inconsistently use contraception	219	7.3
	2. Not use contraception	2149	71.4
In terms of becoming a mother	0. Wrong time	629	20.9
	1. An OK time but not quite right	145	4.8
	2. Right time	2236	74.3
Just before falling pregnant	0. Not intend to become pregnant	734	24.4
	1. Did not mind either way	198	6.6
	2. Intend to get pregnant	2078	69.0
Just before falling pregnant	0. Not want a baby	729	24.2
	1. Have mixed feelings about having a baby	110	3.7
	2. Want a baby	2171	72.1
Before falling pregnant had you and your husband	0. Never discussed children	601	20.0
	1. Discussed children but no firm agreement	274	9.1
	2. Agreed to pregnancy	2135	70.9
Health actions before falling pregnant	0. No Action	1001	33.3
	1. Action	1267	42.1
	2 or more Actions	742	24.7
Total		3010	100.0

## Discussion

In our study the estimated prevalence of unintended pregnancies in women attending the antenatal care clinic was 38.2% which is consistent with the estimated global prevalence [1]. This estimate is higher than previously reported data of 16% and 24% in PDHS 2006 and 2013 [10, 11] but lower than the 46% reported in the study conducted by Sathar et al. [12]. These previous studies used a dichotomous scale whereas we employed the six item LMUP [17–30, 32]. The prevalence of unintended pregnancies in our study is higher than the studies from Iran (33.7%) [21], Kenya (24%) [33], Ethiopia (27.9%) [34] and Sudan (30.2%) [35] but lower than in Nepal (41%) [36], Papua New Guinea (49.4%) [20], Tanzania (45.9%) [37] and Ghana (70%) [38]. The most relevant comparable data are those from Iran and Papua New Guniea [20, 21] who also used the LMUP, although in Papua New Guinea a five item partial LMUP was used as item 6 was dropped to be locally appropriate.

Our study showed that the likelihood of unintended pregnancies is significantly associated with age less than 20 years. This is consistent with the Papua New Guinean, Kenyan and Tanzanian data [20, 33, 37] and makes sense given that younger women have higher fertility, higher frequency of sexual intercourse, lower knowledge of contraceptive methods and higher rates of contraceptive failure relative to older women [33, 37, 39, 40]. Likewise women who were illiterate were more likely to have an unintended pregnancy [20, 35, 36] which is consistent with evidence documenting that literate women have a better understanding of their rights and responsibilities and have more freedom, control and participation in decisions around contraception use and family planning [41–44].

In our study parity was significantly associated with unintended pregnancies. Women who had a parity of greater than two were more likely to have an unintended pregnancy. This finding is comparable to studies conducted in other developing countries [20, 34, 35, 38, 45]. Similar to parity, short birth intervals of less than 12 months were also found to be significantly associated with unintended pregnancies in our study as has been noted elsewhere [20, 46, 47].

Consistent with the available literature [20, 33–38], our study found that unintended pregnancy is strongly associated with a lack of awareness of contraceptive methods. As contraceptive awareness has been found to be directly related to its’ use [48–50] it is essential to implement initiatives to improve community knowledge about contraceptive methods. Our study also found that women who had never used contraception had twice at risk of having an unintended pregnancy compared to current users, as is consistent with the literature [20, 33–38, 51]. Furthermore, ever use of modern methods (including condoms, pills, IUDs, injectables, implants, male and female sterilization and emergency contraception) was very low and use of traditional methods high (Table 4).

This use of modern methods is alarmingly low, particularly the long acting reversible contraceptives (LARC) such as intrauterine devices (IUDs) and implants, possibly due to fear of infertility and side effects [52, 53]. Our data is consistent with the recent PDHS which estimated the unmet need to be 20.1% [11, 54] which is well below that of neighboring countries like India, Nepal and Bangladesh [55–57]. It is evident that National family planning programs are failing to reach many women in need of contraception [52, 58]. Studies from Pakistan have demonstrated that lack of spousal communication, religious beliefs, concerns about infertility and side effects and supply side factors such as poor access, lack of counseling and insufficient availability of modern methods are the major hurdles to the acceptance of modern contraceptive methods [59–61]. Antenatal and postnatal counseling programs in other countries have demonstrated they can improve contraceptive prevalence [62–65]. Similar initiatives could easily be integrated into the Pakistan lady health workers (LHW) program (which has a workforce of more than 100,000 LHWs) [66]. Of course increased availability of modern methods of contraception would need to accompany any such educational program.

After adjusting for other factors women living in rural areas exhibited increased odds of an unintended pregnancy compared to their urban counterparts, a finding consistent with previous studies in similar settings [37, 67, 68], this is likely to be associated with the higher prevalence of poverty, illiteracy, poor contraceptive knowledge and little access to modern contraceptive methods and services in rural areas. Additionally rural women may not have autonomy in decision making and may have little or no say in family planning decisions [68, 69].

Our results indicate that the Urdu LMUP in Pakistan performed very well, with demonstrated reliability and validity in terms of acceptability, targeting, internal consistency and structural validity. The validation results are comparable (Table 7) with the similar validation studies for LMUP conducted in Iran, Malawi, India, United States and Brazil [21–23, 26, 30].

The use of a validated pregnancy intention scale (LMUP) to estimate unintended pregnancies was the main strength of our study, but there are some limitations. Firstly there is a possibility of recall bias due to retrospective nature of the questionnaire. Secondly the cross-sectional design does not allow causal inferences and lastly the results may not be generalizable to the whole country since the study was conducted only in two tertiary care hospitals. And finally is the lack of a test-retest analysis in the psychometric data for the stability of the scores.

Although efforts are being made by both private and public institutions, access to modern methods remains a challenge in Pakistan. Similarly the provision of safe abortion services remained a neglected area due to its illegal status and stigmatization [70]. Recent estimates suggest that about 25,000 unintended pregnancies and their related abortions and unplanned births could be averted over a 5-year period only by changing 4% of current oral contraceptive users in Pakistan to LARC [71]. Community midwives and lady health visitors are well placed to provide LARC services [52] that will allow women the possibility of birth spacing and family limiting [72]. As many women in Pakistan do not have the freedom to decide about family planning it is essential that men are also engaged in education programs which have been found to effectively improve attitudes and behaviors, a decrease in the fertility and an increase in the contraceptive use [73].

## Conclusion

The high prevalence of unintended pregnancies resulting in induced abortions and unplanned births in Pakistan highlight the urgent need for a concerted effort through a private and public partnership to improve the knowledge and access to modern contraceptive methods and safe abortion services. An integrated national family program that provides contraceptive services to women during pre-conception and post-partum would be beneficial in averting unintended pregnancies and their related adverse outcomes in Pakistan.

## Abbreviations

IUD: Intra uterine device
LARC: Long acting reversible contraceptives
LHW: Lady health worker
LMUP: London measure of unplanned pregnancies
PDHS: Pakistan demographic and health survey
